# Per and polyfluoroalkyl substances affect thyroid hormones for people with a history of exposure from drinking water

**DOI:** 10.1038/s41598-025-91977-y

**Published:** 2025-04-11

**Authors:** Taylor S. Noyes, Laura M. Abington, T. Joost van ‘t Erve, Ling Wang, Jennifer M. McDonald, Elizabeth A. Wasilevich, Jennifer S. Gray, Timothy A. Karrer, Kristine Smith, Jordan M. Bailey

**Affiliations:** 1 Michigan Department of Health and Human Services, Environmental Health Bureau, 333 South Grand Ave., 3rd Floor, Lansing, MI 48909 USA; 2 Department of Medicine, College of Human Medicine, Michigan State University, East Lansing, MI USA; 3 Division of Chemistry and Toxicology, Michigan Department of Health and Human Services, Bureau of Laboratories, Lansing, MI USA; 4 Division of Infectious Disease, Michigan Department of Health and Human Services, Bureau of Laboratories, Lansing, MI USA

**Keywords:** Per- and polyfluoroalkyl substances, PFAS, Epidemiology, Endocrine disruption, Thyroid hormone, Mixture analysis, Biomarkers, Diseases, Epidemiology

## Abstract

Per- and polyfluoroalkyl substances (PFAS) may disrupt thyroid hormones although the literature shows mixed evidence of this effect and exposure to mixtures of PFAS remains poorly understood. We used the Michigan PFAS Exposure and Health Study cohort to examine linear and nonlinear associations between serum PFAS concentrations, both alone and as a mixture, and serum thyroid hormone concentrations. Study participants included 728 adolescents and adults living in an area with past PFAS contamination of drinking water. We quantified 39 individual PFAS and four thyroid hormones in serum from participants between the years 2020 and 2021. Linear regression, weighted quantile sum (WQS) regression, supervised Principal Component Analysis (PCA), and Bayesian Kernel Machine Regression (BKMR) were used. When analyzed individually, a 1% increase in PFUnA serum concentrations was associated with a 0.023% decrease in TT3 concentration (95% CI: -0.04%, -0.01%, *p* < 0.05). All three mixture analyses consistently indicated an inverse relationship between PFAS mixtures and TT3 concentrations: (1) a one standard deviation increase in the WQS of the PFAS mixture was associated with a 2.0% decrease in TT3 concentration (95% CI= -4%, 0%, *p* < 0.05) adjusting for covariates, (2) using PCA, one standard deviation increase in a PFAS mixture was associated with a 1.2% decrease in TT3 (95% CI: -2.1%, -0.4%), and (3) BKMR similarly suggested a negative association between the PFAS mixture and TT3. We observed cross-sectional associations between a mixture of serum PFAS concentrations and thyroid hormone dysregulation, largely manifesting as decreased TT3 serum concentrations.

**Supplementary Information:**

The online version contains supplementary material available at 10.1038/s41598-025-91977-y.

## Introduction

Per- and polyfluoroalkyl substances (PFAS) are a family of thousands of human-made chemicals with wide industrial and consumer use. The general population is exposed to PFAS through food, drinking water, consumer products, and household dust^1–5^. Although limited to a relatively small subset of the thousands of known PFAS, data from both the toxicological and epidemiological literatures have consistently linked exposure to PFAS with numerous health effects^1,6^. Endocrine disruption^7^, including specific targets within the thyroid system, have been suggested^8^ among the myriad consequences associated with exposure to PFAS.

Evidence that PFAS exposure may be associated with thyroid hormone dysfunction during pregnancy has accumulated^9–12^ although both negative and positive correlations have been reported^13–19^. The impact of PFAS on thyroid hormones outside of pregnancy is similarly equivocal. Among non-pregnant adults, no clear pattern of effects emerges among either general or highly PFAS-exposed populations on outcomes such as free/total triiodothyronine (fT3/TT3), free/total thyroxine (fT4/TT4), and thyroid stimulating hormone (TSH)^20,21^. These inconsistent findings may reflect effects that are dependent on unique characteristics of the population, the timeframe covered by the analyses, or the unique mixtures of PFAS encountered among the population studied. Mechanistically, various PFAS have been shown to alter thyroid hormone synthesis in several ways, including inhibition of Na+/I- symporter-mediated iodide uptake and reduction of thyroid peroxidase levels^22,23^ also see commentary by^24^.

Further complicating our understanding of the role of PFAS in thyroid hormone disruption is the poorly characterized importance of human exposure(s) to numerous and varied PFAS mixtures. Exposure to multiple PFAS likely represents the most relevant human exposure paradigm, and resultant health effects will likely need to be understood within a broad exposome context inclusive of other environmental toxicants. Like the effects of individual PFAS on thyroid hormones, the results of PFAS mixture analyses on endpoints related to thyroid function (namely total/free thyroxine (T_4_), total/free triiodothyronine (T_3_) and thyroid stimulating hormone (TSH)), have been inconsistent^9,14,25,26^.

In 2017–2018, two communities in western Michigan were found to have high concentrations of PFAS mixtures in drinking water. The Michigan Department of Health and Human Services (MDHHS), alongside other state and local agencies, worked to dramatically reduce their exposure to PFAS via drinking water. PFAS from industrial sources at nearby landfills is thought to have impacted the ground water used for drinking water in these communities. Exposure is presumed to have begun decades before discovery, based on available information about the operation of these landfills, however, precise onset of contamination is not known. Given their prolonged and high-concentration exposure to a mixture of PFAS, individuals from these communities were recruited to participate in the Michigan PFAS Exposure and Health Study (MiPEHS)^27,28^ which is a longitudinal study that aims to understand what relations exist among various health endpoints and biomarkers of PFAS exposure. MiPEHS has previously demonstrated elevated serum PFAS concentrations in the study population^27,28^.

Our aim for the present study was to evaluate the relationship of PFAS, both individually and as a mixture, with serum thyroid hormone concentrations among a highly exposed non-pregnant population. Our selection of a population who experienced prolonged and high-concentrations of multiple PFAS in drinking water makes the identification of associations between thyroid hormones and serum PFAS concentrations more likely, if a dose-response relationship is assumed, compared to examinations of the general population.

## Methods

### Study design and population

From December 2020 to August 2021, we recruited 1,054 individuals into the first phase of the longitudinal Michigan PFAS Exposure and Health Study (MiPEHS), based in Michigan, USA. To be eligible for MiPEHS, participants were required to have lived in either of two communities impacted by PFAS-contaminated drinking water between 2005 and 2018. Because extensive response (e.g., emergency response, water provisioning, water sampling) activities had already occurred in these areas, impacted residences were known. A total of 5,969 potentially eligible residences were recruited by direct mail, phone call, door knocking and indirect advertisement (e.g., social/print media, local events). All participants completed a web-based survey and attended a visit to a local study office where we collected blood samples and took body measurements. Of an estimated total recruited population of 14,922 (i.e., number of potentially eligible residences multiplied by estimated number of people per residence (2.5)), 1,054 people joined MiPEHS, of which 919 provided blood samples that were analyzed for PFAS and thyroid hormones. Participants were excluded from thyroid hormone statistical analyses if they were pregnant (*n* = 4), reported taking thyroid medications within the last 30 days (*n* = 122), or had missing values for any of the model covariates such as sex, age (*n* = 65). The thyroid statistical analyses, therefore, included 728 adolescents (aged 12 to 19 years) and adults (aged over 20 years).

All study participants provided written informed consent before participation. All aspects of the study were approved by the Michigan Department of Health and Human Services (MDHHS) Institutional Review Board (MDHHS IRB Log #202003-03-FC). All aspects of the study were conducted in accordance with the MDHHS IRB.

### Quantification of serum PFAS concentrations

We measured the concentration of 39 PFAS in serum separated from clotted venous blood samples collected during participant’s study office visit (collected in red top tubes). All serum samples were shipped on dry ice and stored at or below − 80 °C until analysis. All PFAS analyses were performed at the Michigan Department of Health and Human Services Bureau of Laboratories (MDHHS BOL). Prior to analysis, sample preparation entailed isotope dilution and the addition of acetonitrile to precipitate proteins. Samples were further cleaned using a 96-well filtration plate and concentrated 20-fold prior to analysis. Sample preparation and analytical measurements were conducted using a validated method and followed strict quality control and quality assurances in accordance with College of American Pathologist (CAP) and Clinical Laboratory Improvement Amendments (CLIA) regulations. Branched isomers of PFOS, PFOA and PFHxS, identified below with “Br” (branched) and “Ln” (linear) prefixes, were quantified. Native and isotopically-labelled standards were purchased from Wellington Laboratories Inc, Guelph, Ontario, Canada. Analyses were performed using Shimadzu LC-MS 8060 mass spectrometers. The full technical details of this method will be published separately. Supplemental Table S1 includes the full list of PFAS measured with their corresponding limit of quantification (LOQ).

### Quantification of serum thyroid hormone concentrations

We measured TSH, fT4, TT3 and TT4. Of those, all the TSH, fT4, and TT4 analyses were conducted at the MDHHS BOL. These thyroid hormones were measured in serum separated from clotted venous blood samples collected during the participant’s study office visit (collected in gold top tubes) using Dimension^®^ 200 EXL™ (with LOCI^®^ Module) automated immunoassays (Siemens Healthcare Diagnostics). Samples were assayed in random order and included internal system checks and external quality control samples as required by the analyzer and laboratory procedures. Most serum TT3 measurements (87%) were performed by Mayo Clinic Laboratories^29^, using a Roche triiodothyronine assay (T3) competitive assay using electrochemiluminescence detection. A minority (13%) of the TT3 measurements were performed at the MDHHS BOL, using ADVIA Centaur XPT competitive immunoassays using direct chemiluminescent technology (Siemens Healthcare Diagnostics). No statistically significant differences were observed between samples quantified at Mayo versus those quantified in-house, and the datasets were combined for all further analyses. All reference ranges are reported in Supplemental Table S2.

### Covariates

Self-reported participant characteristics were available from the survey, which included sex, gender, age, prior diagnosis of health conditions, family health history, drinking water habits, residential history, extended health and family health history, lifestyle factors including smoking status and alcohol use and potential sources of PFAS exposure other than drinking water. Participants reported drinking water habits for the past (before they know about PFAS contamination) and the present. A trained study staff member took body measurements including height, weight, hip and waist circumference and blood pressure.

### Statistical analyses

We computed descriptive statistics for demographic characteristics, PFAS and thyroid hormone concentrations, and used Pearson correlation coefficients to determine the correlation among log-transformed PFAS (Supplemental Figure S1). Values for PFAS reported as non-detect (ND) or below the limit of detection (LOD) were recoded to $\:\frac{limit\:of\:quantification}{\sqrt{2}}$ and $\:\frac{limit\:of\:detection}{\sqrt{2}}$, respectively. All analyses were performed using natural log (ln) transformed PFAS, TSH, fT4, and TT3 values to meet normality assumptions. TT4 concentrations were normally distributed in their original form and therefore did not require transformation. Two participants with a TSH result greater than 20 µIU/L were excluded from the TSH models in order to satisfy the normal distribution assumption. Twelve PFAS had a detection frequency of 60% or greater and were used in regression models (Supplemental Table S1).

In addition to a linear regression model, we also selected several methods that intentionally model mixture exposures and permit us to understand PFAS mixture effects on thyroid hormones: weighted quantile sum (WQS) regression, supervised principal component analysis (PCA), and Bayesian kernel machine regression (BKMR).

Covariates retained in the adjusted models were selected following a literature review and the construction of a directed acyclic graph (DAGgity 3.0, Nijmegen, NL)^30^ which was used to identify the minimal adjustment set of potentially confounding factors. Selected covariates included self-reported sex, age at the date of blood draw, current smoking status, recent alcohol use, and family history of thyroid disease. To aid in the interpretation of the estimated intercept, we used mean-centered age in the analysis so that the intercept represents the thyroid function of the individual at mean age in the study sample. For BKMR analyses, PFHpS was removed from the analyses to avoid multicollinearity due to its strong correlation with other PFAS, such as PFHxS, PFOA and Br-PFOS (correlation coefficients (ρ) = 0.89, 0.84 and 0.9, respectively, Supplemental Figure S1).

Data management, descriptive analyses and linear regression were conducted using SAS 9.4 (SAS Institute, Cary, NC). Mixture analyses were conducted with R version 4.0.4 (R Core Development Team) using R packages “gWQS”, “superpc”, and “bkmr”.

#### Linear regression

To understand the effect of PFAS serum concentrations on thyroid hormones, we first fit linear regression models for each thyroid hormone and each PFAS separately, adjusting for covariates and correcting for multiple comparisons. This analysis estimated a beta coefficient and 95% confidence internal (CI) for each PFAS, which was back-transformed to percent change in thyroid hormone concentrations as PFAS concentrations increased by 1%. This was done by using the equations $\:(\text{e}\text{x}\text{p}(\widehat{\beta\:})-1)*100$ for TSH, fT4, and TT3 (where both the dependent and independent variables were log-transformed) and $\:\widehat{\beta\:}*log\:\left(1.01\right)\:$ for TT4 (where only the independent variable was log-transformed), where $\:\widehat{\beta\:}$ is the estimate from each linear regression model. The Benjamini-Hochberg (BH) correction was performed on p-values to adjust for the multiple testing problem in linear regression. Both BH adjusted P-values and original P-values are reported.

#### Weighted quantile sum (WQS) regression

We used WQS regression to understand the effect of a PFAS mixture on thyroid hormones and to identify the driving components of that mixture. WQS permits the weighting of the association between each PFAS and the outcome, and assesses them simultaneously using a weighted additivity index, providing an overall mixture effect estimate^31^. The study sample was randomly split into a training dataset (40%, *n* = 291) and a validation data set (60%, *n* = 437). The exposure was first scored into deciles in training data set and overall quantile scores was created by summing the deciles in the training data set. Weights for WQS score were estimated using 10 bootstrap samples from training data set. The weights were then used to create a WQS index representing the overall mixture of PFAS. The final significance of the WQS index were tested in the validation data (*n* = 437) for all four thyroid function measurements. For this analysis, PFAS with estimated weights > 0.08 (1/12) were considered to appreciably contribute to the WQS index, as their weights were over the average weight of the 12 PFAS.

#### Supervised principal components analysis (sPCA)

We used supervised PCA to reduce the 12 PFAS measurements into a smaller subset of uncorrelated principals components^32,33^. Supervised principal components analysis is a variation of principal components regression with feature selection at the first stage. The principal components are the linear combinations of the features that capture the directions of largest variation in a dataset. To identify linear combinations related to a thyroid hormone, coefficients from univariate analysis for each PFAS analyte were calculated and only those PFAS whose coefficients exceeded a threshold were retained in the PCA analysis. The cutoff thresholds of coefficients were 0.55, 0.5, 0.6 and 0.4 for log-transformed fT4, log-transformed TSH, log-transformed TT3, and TT4, respectively.

#### Bayesian kernel machine regression (BKMR)

BKMR was also used to evaluate potential PFAS mixture effects on thyroid hormones because it uses a nonparametric approach to identify the relationship between PFAS and thyroid hormones while allowing for nonlinear associations^34^. BKMR allowed us to quantify potential nonlinearity and non-additive effects of a PFAS mixture and to identify PFAS analytes primarily responsible for the overall effect by comparing posterior inclusion probabilities (PIPs) and detecting interactions between mixture components. The model specification for Bayesian Kernel Machine Regression (BKMR) in our analysis was:

$${Y_i}=h\left( {{Z_{i1}}, \ldots ,{Z_{iM}}} \right)+\beta {X_i},~i=1, \ldots ,n$$

Where $\:{Y}_{i}$ is the thyroid hormone level for each individual (log transformed fT4, TSH and TT3, and original/non-transformed TT4 concentration), $\:{Z}_{i1},\dots\:,{Z}_{iM}$ are the individual PFAS concentrations of individual $\:i$, and $\:{X}_{i}$ are covariates. $\:h$ is a flexible function of PFAS for which nonlinear and non-additive relationships are assumed. Specifically, $\:h\left({Z}_{i1},\dots\:,{Z}_{iM}\right)={\sum\:}_{j=1}^{M}K({Z}_{j},\:\:Z){\alpha\:}_{j}$ and $\:K$ is the Gaussian Kernel.

## Results

Participant characteristics, serum PFAS, and biomarkers of the 728 adolescents and adults included in the analyses are shown in Table 1. Participants ranged in age from 12 years to 100 years, with a median age of 56 years. Most participants were non-Hispanic white (92.2%), and equal numbers of males and females were represented. About a quarter of the participants reported a known family history of thyroid disease (22.1%) and over half (61%) reported current alcohol use.

The twelve PFAS detected in at least 60% of serum samples and selected for further analyses included: MeFOSAA (N-Methylperfluorooctane sulfonamidoacetic acid), PFDA (Perfluorodecanoic acid), PFHpA (Perfluoroheptanoic acid), PFHpS (Perfluoroheptanesulfonic acid), PFNA (Perfluorononanoic acid), PFPeS (Perfluoropentanesulfonic acid), PFUnA (Perfluoroundecanoic acid), PFECHS (Perfluoroethylcyclohexane sulfonate), PFOA (Perfluorooctanoic acid), PFHxS (Perfluorohexanesulfonic acid), and both the branched (Br) and linear (Ln) isomers of PFOS.

**Table 1:** Serum analyte and biomarker concentrations by participant characteristics (*n* = 728).

Participant Characteristics		*N* (%)	Serum PFAS Thyroid Hormones Geometric Mean (95% Confidence Interval) Geometric Mean (95% Confidence Interval)															
			MeFOSAA (µg/L)	PFDA (µg/L)	PFHpA (µg/L)	PFHpS (µg/L)	PFNA (µg/L)	PFPeS (µg/L)	PFUnA (µg/L)	PFECHS (µg/L)	PFOA (µg/L)	Br_PFOS (µg/L)	Ln_PFOS (µg/L)	PFHxS (µg/L)	TSH (mIU/L)	TT3 (ng/dL)	TT4 (µg/dL)	fT4 (ng/dL)
Total		728 (100)	0.11 (0.1, 0.12)	0.1 (0.09, 0.11)	0.03 (0.02, 0.03)	0.34 (0.3, 0.38)	0.35 (0.33, 0.37)	0.03 (0.03, 0.03)	0.05 (0.04, 0.05)	0.03 (0.03, 0.04)	3.18 (2.82, 3.58)	4.81 (4.36,5.29)	3.9 (3.59,4.22)	1.47 (1.33, 1.62)	1.75 (1.67, 1.83)	111.79 (110.24, 113.36)	7.42 (7.27, 7.57)	0.99 (0.98, 1)
Sex																		
Male		364 (50)	0.12 (0.11, 0.13)	0.11 (0.1, 0.12)	0.03 (0.02, 0.03)	0.46 (0.4, 0.53)	0.38 (0.35, 0.42)	0.03 (0.03, 0.04)	0.05 (0.05, 0.06)	0.04 (0.04, 0.05)	3.45 (2.93, 4.06)	5.87 (5.15,6.69)	4.53 (4.03,5.08)	1.98 (1.74, 2.26)	1.78 (1.69, 1.88)	112.81 (110.7, 114.95)	7.14 (6.94, 7.34)	0.99 (0.98, 1)
Female		364 (50)	0.1 (0.09, 0.12)	0.09 (0.08, 0.1)	0.02 (0.02, 0.03)	0.25 (0.21, 0.29)	0.32 (0.29, 0.35)	0.03 (0.03, 0.03)	0.04 (0.04, 0.05)	0.03 (0.03, 0.03)	2.92 (2.46, 3.47)	3.94 (3.42,4.53)	3.35 (3,3.75)	1.08 (0.94, 1.24)	1.73 (1.6, 1.86)	110.79 (108.53, 113.09)	7.7 (7.48, 7.93)	0.99 (0.98, 1)
Age category																		
12–19		64 (8.8)	0.08 (0.06, 0.1)	0.07 (0.05, 0.08)	0.04 (0.03, 0.05)	0.18 (0.13, 0.25)	0.21 (0.19, 0.24)	0.04 (0.03, 0.05)	0.03 (0.02, 0.03)	0.02 (0.02, 0.02)	2.77 (1.97, 3.89)	2.94 (2.23,3.88)	5.75 (4.39, 7.53)	0.81 (0.6, 1.1)	1.73 (1.47, 2.04)	139.99 (132.55, 147.85)	7.71 (7.34, 8.1)	1 (0.97, 1.04)
20–39		101 (13.9)	0.08 (0.06, 0.09)	0.07 (0.06, 0.08)	0.02 (0.02, 0.02)	0.22 (0.16, 0.31)	0.24 (0.2, 0.28)	0.03 (0.02, 0.03)	0.03 (0.03, 0.04)	0.03 (0.02, 0.04)	2.9 (2.11, 4)	3.71 (2.83, 4.85)	3.03 (2.42,3.8)	1.13 (0.84, 1.53)	1.52 (1.32, 1.76)	116.91 (112.32, 121.69)	7.51 (7.04, 8.02)	1 (0.98, 1.02)
40–59		239 (32.8)	0.09 (0.08, 0.11)	0.1 (0.09, 0.11)	0.02 (0.02, 0.03)	0.29 (0.24, 0.35)	0.34 (0.31, 0.37)	0.03 (0.02, 0.03)	0.05 (0.04, 0.06)	0.03 (0.03, 0.04)	2.96 (2.41, 3.62)	4.31 (3.63, 5.11)	3.6 (3.14, 4.12)	1.28 (1.06, 1.54)	1.72 (1.62, 1.83)	109.69 (107.53, 111.89)	7.26 (6.98, 7.54)	0.97 (0.96, 0.99)
60+		324 (44.5)	0.15 (0.14, 0.17)	0.12 (0.11, 0.13)	0.03 (0.02, 0.03)	0.49 (0.42, 0.56)	0.44 (0.4, 0.49)	0.03 (0.03, 0.04)	0.06 (0.05, 0.07)	0.04 (0.04, 0.05)	3.54 (2.94, 4.26)	6.17 (5.37, 7.09)	4.85 (4.32, 5.46)	1.97 (1.74, 2.23)	1.86 (1.73, 2)	107.03 (105.03, 109.07)	7.45 (7.24, 7.65)	1 (0.98, 1.02)
Race and ethnicity																		
Hispanic Black, Indigenous, or person of color (BIPOC)		6 (0.8)	0.14 (0.04, 0.51)	0.08 (0.05, 0.14)	0.04 (0.01, 0.14)	0.77 (0.36, 1.62)	0.34 (0.23, 0.5)	0.07 (0.02, 0.31)	0.03 (0.01, 0.1)	0.04 (0.03, 0.06)	6.34 (2.41, 16.69)	6.89 (3.69,12.86)	4.53 (2.8,7.32)	2.55 (0.9, 7.25)	1.6 (0.99, 2.58)	131.55 (121.61, 142.31)	9.08 (7.95, 10.37)	1.02 (0.87, 1.2)
Hispanic white		7 (1)	0.07 (0.03, 0.15)	0.05 (0.02, 0.09)	0.02 (0.01, 0.03)	0.21 (0.06, 0.7)	0.14 (0.05, 0.4)	0.03 (0.02, 0.05)	0.02 (0.01, 0.03)	0.02 (0.01, 0.03)	2.66 (0.69, 10.2)	3.29 (1.14,9.48)	1.92 (0.95,3.84)	1.07 (0.42, 2.73)	1.31 (1.01, 1.7)	106.75 (92.44, 123.27)	7.96 (6.94, 9.13)	0.98 (0.9, 1.06)
Non, Hispanic Black, Indigenous, or person of color (BIPOC)		37 (5.1)	0.08 (0.06, 0.12)	0.12 (0.09, 0.16)	0.02 (0.02, 0.03)	0.57 (0.37, 0.88)	0.41 (0.33, 0.52)	0.04 (0.02, 0.06)	0.05 (0.04, 0.08)	0.03 (0.03, 0.05)	7.44 (4.36, 12.69)	5.42 (3.85,7.63)	13.58 (9.35, 19.72)	2.5 (1.58, 3.98)	1.52 (1.24, 1.86)	111.79 (104.7, 119.37)	7.14 (6.33, 8.05)	1.01 (0.98, 1.05)
Non, Hispanic white		671 (92.2)	0.11 (0.1, 0.12)	0.1 (0.09, 0.11)	0.03 (0.02, 0.03)	0.33 (0.29, 0.37)	0.35 (0.33, 0.38)	0.03 (0.03, 0.03)	0.05 (0.05, 0.05)	0.03 (0.03, 0.04)	3.06 (2.7, 3.45)	4.71 (4.26,5.21)	3.9 (3.59,4.23)	1.43 (1.3, 1.59)	1.77 (1.69, 1.86)	111.69 (110.08, 113.33)	7.41 (7.25, 7.57)	0.99 (0.98, 1)
Not specified		7 (1)	0.1 (0.04, 0.3)	0.06 (0.03, 0.14)	0.02 (0.01, 0.05)	0.14 (0.04, 0.45)	0.16 (0.05, 0.51)	0.03 (0.01, 0.08)	0.03 (0.01, 0.06)	0.03 (0.01, 0.06)	0.94 (0.2, 4.5)	1.67 (0.41,6.75)	1.15 (0.31,4.33)	0.62 (0.2, 1.9)	1.78 (1.45, 2.19)	110.12 (100.62, 120.52)	7.8 (6.85, 8.89)	0.98 (0.91, 1.07)
Community																		
City of Parchment Municipal Supply		211 (29)	0.09 (0.08, 0.11)	0.09 (0.08, 0.1)	0.03 (0.02, 0.03)	0.97 (0.81, 1.15)	0.4 (0.36, 0.44)	0.04 (0.04, 0.05)	0.04 (0.03, 0.04)	0.04 (0.03, 0.04)	17.87 (14.65, 21.79)	15.33 (12.9,18.22)	7.69 (6.67,8.87)	2.38 (2.04, 2.77)	1.65 (1.52, 1.79)	115.85 (113.19, 118.56)	7.46 (7.19, 7.74)	0.99 (0.98, 1.01)
Cooper Township Private Wells		126 (17.3)	0.12 (0.1, 0.15)	0.09 (0.08, 0.11)	0.02 (0.02, 0.03)	0.22 (0.18, 0.26)	0.29 (0.25, 0.34)	0.02 (0.01, 0.02)	0.05 (0.04, 0.06)	0.02 (0.02, 0.03)	2.01 (1.67, 2.43)	3.35 (2.92,3.86)	2.77 (2.39,3.21)	1.22 (1.01, 1.48)	1.85 (1.7, 2.01)	112.68 (108.61, 116.9)	7.43 (7.01, 7.87)	0.99 (0.97, 1.01)
Belmont/ Rockford Private Wells		391 (53.7)	0.12 (0.11, 0.13)	0.11 (0.1, 0.12)	0.03 (0.02, 0.03)	0.22 (0.19, 0.25)	0.35 (0.32, 0.38)	0.03 (0.03, 0.03)	0.05 (0.05, 0.06)	0.04 (0.03, 0.04)	1.45 (1.3, 1.61)	2.89 (2.59,3.21)	3.03 (2.73,3.37)	1.2 (1.04, 1.38)	1.78 (1.66, 1.9)	109.35 (107.26, 111.47)	7.39 (7.2, 7.59)	0.99 (0.98, 1)
Current alcohol use																		
Yes		442 (60.7)	0.11 (0.1, 0.12)	0.11 (0.1, 0.12)	0.03 (0.02, 0.03)	0.33 (0.29, 0.38)	0.39 (0.36, 0.42)	0.03 (0.03, 0.03)	0.06 (0.05, 0.06)	0.04 (0.04, 0.04)	2.94 (2.52, 3.43)	4.71 (4.15,5.35)	3.42 (3.03,3.86)	1.47 (1.29, 1.68)	1.71 (1.61, 1.8)	107.76 (106.04, 109.52)	7.21 (7.03, 7.41)	0.98 (0.97, 0.99)
No		286 (39.3)	0.11 (0.1, 0.13)	0.08 (0.07, 0.09)	0.02 (0.02, 0.03)	0.34 (0.29, 0.41)	0.3 (0.28, 0.33)	0.03 (0.03, 0.03)	0.04 (0.03, 0.04)	0.03 (0.03, 0.03)	3.58 (2.97, 4.3)	4.9 (4.23,5.68)	4.26 (3.83,4.75)	1.46 (1.26, 1.68)	1.83 (1.69, 1.98)	118.42 (115.63, 121.29)	7.74 (7.49, 7.98)	1.01 (0.99, 1.02)
Current smoker																		
Yes		39 (5.4)	0.11 (0.08, 0.15)	0.07 (0.06, 0.1)	0.02 (0.02, 0.03)	0.44 (0.25, 0.78)	0.3 (0.23, 0.4)	0.04 (0.02, 0.05)	0.04 (0.03, 0.05)	0.04 (0.03, 0.05)	5.03 (2.64, 9.58)	6.96 (4.19,11.57)	3.94 (2.55,6.07)	1.53 (0.95, 2.48)	1.83 (1.58, 2.12)	117.63 (111.63, 123.96)	7.67 (7.05, 8.33)	0.99 (0.96, 1.03)
No		689 (94.6)	0.11 (0.1, 0.12)	0.1 (0.1, 0.11)	0.03 (0.02, 0.03)	0.33 (0.3, 0.37)	0.35 (0.33, 0.38)	0.03 (0.03, 0.03)	0.05 (0.05, 0.05)	0.03 (0.03, 0.04)	3.09 (2.74, 3.49)	4.71 (4.27,5.2)	3.9 (3.59,4.23)	1.46 (1.32, 1.61)	1.75 (1.67, 1.83)	111.47 (109.87, 113.09)	7.4 (7.25, 7.56)	0.99 (0.98, 1)
Family history of thyroid disease																		
Yes		161 (22.1)	0.12 (0.1, 0.14)	0.1 (0.09, 0.12)	0.02 (0.02, 0.03)	0.29 (0.23, 0.36)	0.33 (0.29, 0.38)	0.02 (0.02, 0.03)	0.05 (0.04, 0.06)	0.03 (0.03, 0.04)	2.5 (1.97, 3.18)	4.06 (3.35,4.9)	3.67 (3.16,4.26)	1.3 (1.06, 1.6)	1.69 (1.5, 1.91)	112.79 (109.31, 116.37)	7.55 (7.23, 7.89)	0.99 (0.97, 1.01)
No		567 (77.9)	0.11 (0.1, 0.12)	0.1 (0.09, 0.11)	0.03 (0.02, 0.03)	0.35 (0.31, 0.4)	0.35 (0.33, 0.38)	0.03 (0.03, 0.04)	0.05 (0.04, 0.05)	0.03 (0.03, 0.04)	3.4 (2.97, 3.89)	5 (4.48,5.59)	3.97 (3.62,4.37)	1.52 (1.36, 1.69)	1.77 (1.69, 1.86)	111.51 (109.78, 113.26)	7.38 (7.21, 7.55)	0.99 (0.98, 1)

Notes. The City of Parchment and Cooper Township are two locations within the same overall “community.” N-Methylperfluorooctane sulfonamidoacetic acid (MeFOSAA); Perfluorodecanoic acid (PFDA); Perfluoroethylcyclohexane sulfonate (PFECHS); Perfluoroheptanoic acid (PFHpA); Perfluoroheptanesulfonic acid (PFHpS); Perfluorononanoic acid (PFNA); Perfluoropentanesulfonic acid (PFPeS); Perfluoroundecanoic acid (PFUnA); Perfluorohexanesulfonic acid) (PFHxS); Perfluorooctanoic acid (PFOA); Perfluorooctanesulfonic acid (Br: branched, Ln: linear) (PFOS); Thyroid Stimulating Hormone (TSH); Total Triiodothyronine (TT3); Free Thyroxine (fT4); Total Thyroxine (TT4).

### Single PFAS models: linear regression

We found that for each 1% increase in PFUnA serum concentrations, TT3 levels decreased by 0.023% (95% CI: -0.04%, -0.01%, adjusted *p* < 0.05) after adjusting for multiple comparisons and controlling for covariates. No other single PFAS and thyroid hormone association was statistically significant after adjusting for multiple comparisons (Supplemental Table S3).

### PFAS mixture models: weighted quantile sum (WQS) regression

The WQS index was associated with decreased serum TT3 concentrations. A one standard deviation increase in the WQS of the PFAS mixture was associated with a 2.0% (95% CI= -4%, 0%, *p* < 0.05) decrease in TT3 concentration adjusting for covariates (Table2). The individual PFAS weights in the WQS index for TT3 were: PFUnA (34%), PFECHS (26%), PFPeS (21%), PFHpA (8%), PFHxS (7%), and PFDA (5%) (see Supplemental Figure S2). The results of using WQS to examine any positive relationships among the mixture and thyroid hormones revealed no significant effects.

**Table 2:** Adjusted weighted quantile sum (WQS) regression estimates of (negative) associations between PFAS mixture exposure and thyroid hormones.

Variable	Thyroid hormones							
	TSH^a^ (*n* = 726)		TT4 (*n* = 728)		fT4^a^ (*n* = 727)		TT3^a^ (*n* = 684)	
	Estimates (95% CI)	*p*-value	Estimates (95% CI)	*p*-value	Estimates (95% CI)	*p*-value	Estimates (95% CI)	*p*-value
Standardized WQS Index^b^	0.02 (-0.04,0.09)	0.47	-0.06 (-0.22,0.09)	0.44	-0.005 (-0.01,0)	0.28	-0.02 (-0.04,0)	0.04
Sex	-0.001 (-0.12,0.12)	0.99	0.35 (0.05,0.65)	0.02	-0.01 (-0.03,0.02)	0.59	-0.04 (-0.08,-0.01)	0.02
Recent cigarette use	-0.002 (-0.28,0.27)	0.99	0.28 (-0.37,0.94)	0.4	-0.03 (-0.09,0.03)	0.31	0.07 (0,0.13)	0.05
Recent alcohol use	-0.1 (-0.22,0.03)	0.13	-0.46 (-0.76,-0.16)	0.003	-0.02 (-0.05,0)	0.06	-0.07(-0.11,-0.04)	< 0.01
Age at blood draw	0.002 (-0.0009,0.006)	0.16	0.001 (-0.0076,0.009)	0.86	0 (-0.0004,0.001)	0.37	-0.003 (-0.0036,-0.002)	< 0.01
Family history of thyroid problems	0.02 (-0.12,0.17)	0.77	0.21 (-0.14,0.56)	0.25	0 (-0.03,0.03)	0.96	0 (-0.04,0.04)	0.97

Notes:* TSH* thyroid stimulating hormone,* TT4* total thyroxine,* FT4* free thyroxine,* TT3* total triiodothyronine,* CI* confidence interval. WQS Index is made up of the following PFAS and weights: PFUnA (34%), PFECHS (26%), PFPeS (21%), PFHpA (8%), PFHxS (7%), PFDA (5%). Estimates are based on a one standard deviation increase in the index. All negative associations between PFAS chemical and Thyroid function are assumed. ^a^Log-transformed. ^b^Standardized WQS Index refers to the weighted sum of PFAS mixtures.

### PFAS mixture models: supervised principal component analysis (sPCA)

The first two principal components accounted for most of the variance in serum PFAS concentrations and both were included in multiple linear regression models to obtain effect estimates. The loadings for the first two principal components for TSH, TT4, fT4 and TT3 are shown in Supplemental Table S4. A high absolute value of loadings describes PFAS that strongly influence the components, and the sign of a loading indicates whether PFAS are positively (+) or negatively (-) correlated with component. For TT3, the first component included PFDA, PFECHS, PFNA, and PFUnA, all of which had negative loadings (-0.55, -0.38, -0.54 and − 0.51 respectively). For TSH, the first component included PFDA, PFHpA, PFNA, and PFUnA, all of which had negative loadings (-0.69, -0.21, -0.56 and − 0.68 respectively). For every standard deviation increase in the first component (which indicates a decrease in the mixture of serum PFAS concentrations) there was a 1.2% increase in TT3 (95% CI 0.4%, 2.1%) and a 4.9% decrease in TSH (95% CI -8.5%, -1.4%) (Table 3). The second component, which included MeFOSAA, PFHpS, PFPeS, and Br-PFOS (loadings = 0.96, 0.03, 0.28 and 0.05), had a significant effect on fT4 such that every standard deviation increase in the second component was associated with a 1.3% decrease in fT4 concentration (95% CI -2.3%, -0.3%) (Table 3 and Supplemental Table S4).

**Table 3:** Associations between adherence to supervised PCA components and thyroid hormones.

	Thyroid hormones							
	TSH^a^ (*n* = 726)		TT4 (*n* = 728)		fT4^a^ (*n* = 727)		TT3^a^ (*n* = 684)	
	Estimates (95% CI)	*p*-value	Estimates (95% CI)	*p*-value	Estimates (95% CI)	*p*-value	Estimates (95% CI)	*p*-value
Component 1^b^	-0.049 (-0.085, -0.014)	0.01	0.029 (-0.056, 0.114)	0.50	-0.005 (-0.015, 0.005)	0.37	0.012 (0.004,0.021)	< 0.01
Component 2^c^	-0.044 (-0.091, 0.002)	0.06	-0.09 (-0.219, 0.039)	0.17	-0.013 (-0.023, -0.003)	0.01	-0.002 (-0.018,0.013)	0.79
Sex	-0.018 (-0.11, 0.073)	0.7	0.546 (0.288, 0.804)	< 0.01	-0.002 (-0.021, 0.018)	0.86	-0.03 (-0.056,-0.003)	0.03
Recent cigarette use	0.072 (-0.129, 0.273)	0.48	0.239 (-0.322, 0.799)	0.40	0.002 (-0.041, 0.045)	0.92	0.058 (0,0.116)	0.05
Recent alcohol use	-0.112 (-0.207, -0.016)	0.02	-0.468 (-0.734, -0.202)	< 0.01	-0.026 (-0.046, -0.006)	0.01	-0.069 (-0.097,-0.042)	< 0.01
Age at blood draw	0.002 (0, 0.005)	0.05	0 (-0.007, 0.007)	0.99	0 (-0.001, 0.001)	0.91	-0.003 (-0.004,-0.002)	< 0.01
Family history of thyroid problems	-0.042 (-0.152, 0.067)	0.45	0.093 (-0.213, 0.398)	0.55	-0.001 (-0.024, 0.023)	0.97	0.013 (-0.019,0.044)	0.43

*TSH* thyroid stimulating hormone,* TT4* total thyroxine,* FT4* free thyroxine,* TT3* total triiodothyronine,* CI* confidence interval

^a^Log-transformed

^b^Component 1 contains the following PFAS: PFDA, PFECHS, PFNA, PFPeS, PFUnA, and PFOS.

^c^Component 2 contains the following PFAS: MeFOSAA, PFHpA, PFHxS, and PFOS.

### PFAS mixture models: bayesian kernel machine regression (BKMR)

**Fig. 1:**
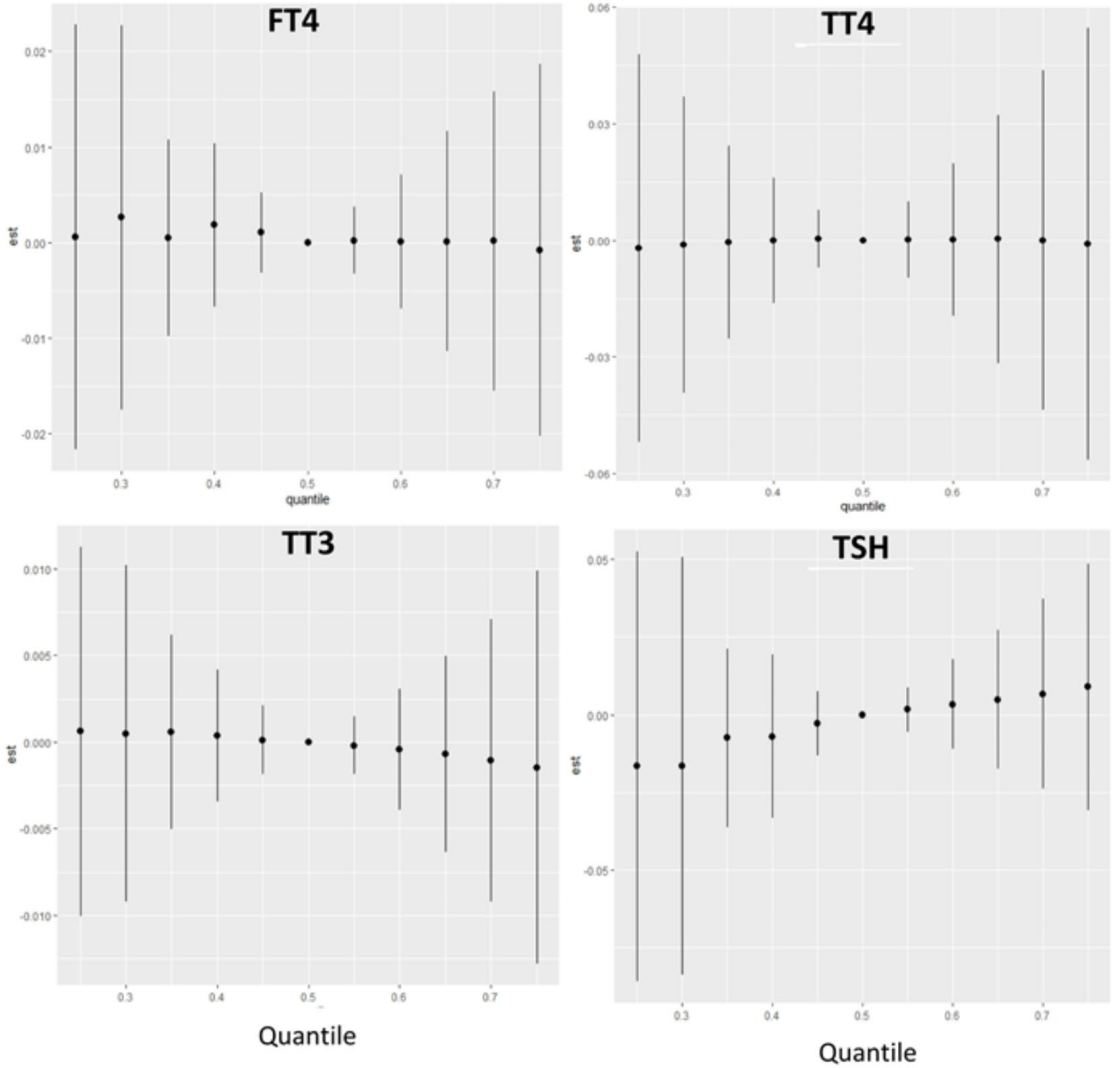
Overall PFAS mixture effect on thyroid hormones from BKMR.

We found a small negative association between the PFAS mixture and TT3, and a small positive association between the PFAS mixture and TSH (Fig. 1). Possible non-linear relationships emerged between the overall mixture effect and thyroid hormone levels, so we explored the exposure-response functions between the interactions of each PFAS and thyroid hormone pair, while other specific PFAS were fixed at defined percentiles (10th, 50th and 90th percentiles) (see Supplemental Figure S3). This indicated possible non-linear exposure-response functions between fT4 and each of the PFAS: MeFOSSA, PFECHS, PFHPA, PFNA, PFUNA, PFPES, PFHXS and PFOA. We also qualitatively examined how these PFAS are interacting with one another related to fT4 concentrations. This shows that PFPES appears to interact with each of the other PFAS in the mixture, with the weakest interaction apparent for PFNA (Supplemental Figure S3).

### Summarized findings from all models

In Table 4, we summarized the top 3 most influential PFAS associated with thyroid hormone levels from all four methods used based on criteria appropriate and relevant to each: (1) linear regression: lowest p-values; (2) WQS regression: highest weights; (3) supervised PCA: highest absolute values of loading; and (4) BKMR: highest values of posterior inclusion probabilities (Supplemental Table S5). Multiple methods (WQS regression, supervised PCA and BKMR) agreed on an inverse relationship between serum PFAS concentrations and serum TT3 concentrations. There was also agreement across methods on which PFAS were driving this relationship. PFUnA was among the top 3 influential PFAS associated with TT3 using WQS, sPCA and BKMR methods and PFDA was among the top 3 influential PFAS associated with TSH using sPCA and BKMR methods.

**Table 4:** Summary of the top 3 PFAS for each method contributing to the relationship with thyroid hormones (TSH, TT4, fT4, TT3) in multiple models in participants of MiPEHS.

PFAS	TSH				TT4				fT4				TT3			
	LR	WQS	sPCA	BKMR	LR	WQS	sPCA	BKMR	LR	WQS	sPCA	BKMR	LR	WQS	sPCA	BKMR
MeFOSAA								^				**✔**				
PFDA		^	^	**✔**			^	^		^					**✔**	**✔**
PFECHS		^				^								**✔**		
PFHpA				**✔**						^		**✔**	**✔**			
PFHpS				NA				NA			**✔**	NA				NA
PFHxS												**✔**				
PFNA			^					^							**✔**	
PFOA																
Ln-PFOS		^				^	^									
Br-PFOS											**✔**					
PFPeS										^	**✔**			**✔**		**✔**
PFUnA			^	**✔**		^	^							**✔**	**✔**	**✔**

Abbreviations: LR: linear regression, WQS: weighted quantile sum regression, sPCA: supervised principal components analysis, BKMR: Bayesian kernel machine regression, TSH: thyroid stimulating hormone, TT4: total thyroxine, FT4: free thyroxine, TT3: total triiodothyronine.

LR “✔“: The checkmark is placed in the cell for the PFAS/thyroid relationship if it ranked among the three lowest significant p-values reported.

WQS “✔“: The checkmark is placed in the cell for the PFAS/thyroid relationship if it ranks among the three highest weights reported for analyses meeting criterion for statistical significance.

sPCA “✔“: The checkmark is placed in the cell for the PFAS/thyroid relationship if it ranks among the three highest absolute loading factors in component 1 for analyses meeting criterion for statistical significance.

BKMR “✔“: The checkmark is placed in the cell for the PFAS/thyroid relationship if it ranks among the top three conditional posterior inclusion probabilities reported for analyses meeting criterion for statistical significance (Supplemental Table S5).

NA: Not included in that analysis

The “^” symbol is placed in the cell for the PFAS/thyroid relationship if it ranks among the top three results for analyses that failed to meet criterion of statistical significance.

## Discussion

This study evaluated the effects of PFAS individually and as a mixture on thyroid hormones among 728 adolescents and adults with a history of exposure to PFAS via their drinking water, which is presumed to have been contaminated for many years. Linear regression of individual PFAS with thyroid hormones showed only one PFAS, PFUnA, that reached criterion for significance. When considering a PFAS mixture, we consistently observed an inverse association between serum PFAS concentrations and TT3 concentrations. We also observed a positive relationship between serum PFAS and TSH concentrations. These findings were robust across methods, with the TT3 effect particularly consistent using three leading-edge mixture modeling approaches (WQS, supervised PCA, and BKMR). Since the changes in TT3 and TSH were relatively small and their absolute values may be understood as subclinical, the implications of these findings at the individual level remain uncertain. However, even small changes in thyroid hormones might have clinical significance considering there are likely people who have borderline thyroid hormone levels where even small changes could result in symptomatic thyroid disease^35^. Although this study was not designed to explore the cellular mechanisms by which PFAS may result in thyroid hormone dysregulation, our results may support the idea that PFAS interferes with the conversion of T4 to T3, explaining why decreases in T3 may be observed even when T4 concentrations remain unaltered^13,36^.

Overall, ambiguities remain in the literature related to which PFAS, or which PFAS mixtures, may have a meaningful impact on thyroid hormone regulation. The timing of exposure to PFAS, the mixture of PFAS involved (or the particular PFAS under study) and the life-stage at which thyroid hormones are quantified are all likely relevant factors to examine. This variability can be observed among studies examining maternal/child cohorts, which have reported positive associations among various PFAS and thyroid hormones^12^, while others report negative associations^10,37,38^, and still others report no effect at all^39^, particularly when TSH is examined^9,39,40^. General population studies or those not emphasizing the maternal/child context are similarly varied, with some reporting positive^41–44^, negative^45^, or null associations^41–44^ between various PFAS and thyroid hormones.

The mixture modeling methods used here allowed us to assess which PFAS are the most important drivers for the mixture effects observed, which, for TT3, consistently suggested PFDA, PFPeS and PFUnA as important contributors. Few studies have comprehensively evaluated the associations between PFAS mixtures and thyroid hormones, and those that have are focused on the maternal/newborn exposure paradigm. The existing literature on the relationship between PFAS mixture exposures and thyroid hormone concentration suggests combined effects of PFAS mixture exposures could contribute to dysfunction within the developing thyroid system; however, the magnitude and direction of these effects and which individual PFAS predominate mixture effects remains equivocal^9,16,39^.

Our study is among the first to evaluate a large array of PFAS both individually and as mixtures while also focusing on a combined adolescent and adult population. Our observed associations are consistent with several previous studies that have found PFDA and PFUnA associated with changes in thyroid hormone concentrations. One study found increasing concentrations of PFDA and PFUnA to be associated with declining concentrations of T3 and fT3^46^ and the other found a similar relationship between PFDA, PFHpA, and PFUnA and TT3^13^. These studies, however, evaluated PFAS individually.

Human exposure to a mixture of environmental contaminants (both between chemical classes and within the class defined as PFAS) is likely the norm, not the exception. Over 60% of our study population had detectable concentrations of 12 individual PFAS, some of which (like PFUnA and PFDA) are relatively less common among the general population^47^. The individual PFAS that contributed most to the mixture effects observed here were found at relatively low concentrations, compared to the concentrations of PFOS and PFOA observed among participants. Despite this, there is support from the literature^13,46^ to suggest these less common PFAS (observed here) may have an important role in mediating PFAS-mixture-induced disruptions to the thyroid system.

In general, our analyses, which employed multiple methods to evaluate a PFAS mixture, yielded consistent and robust findings. These mixture methods are relatively novel approaches for understanding health effects associated with a PFAS mixture, and each has strengths including the ability to handle correlated exposures using composite scores (sPCA, WQS, and BKMR), and model nonlinear effects (BKMR). These methods also have limitations, which are relevant to the current results. Supervised PCA, which selects chemicals based on univariate analysis results in the first step, ignores the correlations among chemicals. WQS assumes an all positive or negative direction of the effects at a time and assessment of bidirectional effects is not possible (although we modelled both directions separately here). Finally, the accuracies of BKMR estimates highly depend on if the convergences are successful or not, which can take large number of iterations to reach.

A few additional limitations of this study are relevant. First, exposure to PFAS in drinking water ended approximately 2 years before we measured serum PFAS concentrations among participants. Therefore, our measurements likely do not represent peak serum concentrations of PFAS, or the same profile as that which may have been present in the past given different half-lives. Despite this, PFAS concentrations above those seen among the general population (as compared to NHANES 1999–2018^47^) were detected among participants. It is possible different observations related to the PFAS mixture and thyroid hormones could have been observed if examined earlier in the exposure. Second, we excluded participants with overt or diagnosed thyroid disease who had recently taken thyroid medication(s). This was necessary to avoid capturing medication-induced thyroid hormone changes, but it means we could have excluded the population with the most aberrant thyroid levels following PFAS exposure. Finally, it is possible our results are subject to multiple testing considerations, although we did explicitly correct for multiple comparisons when needed, and the WQS and BKMR methods control for multiple comparisons inherently.

Our comprehensive evaluation of PFAS on thyroid hormones among a highly exposed population found significant associations between a PFAS mixture and thyroid function. TT3 appeared particularly sensitive to the effects of the PFAS mixture, and mixture modeling methods agreed on the negative association between PFAS mixture and TT3 concentrations. Our results also revealed the potential critical influence of PFDA and PFUnA on the effects of exposure to a mixture of PFAS. Future studies in this population are planned to further investigate the impact of PFAS on thyroid hormones as a function of time, and additional analyses are ongoing within this MiPEHS cohort to understand the occurrence and relationship of clinical thyroid outcomes (e.g., diagnosed thyroid disease) with PFAS serum concentrations.

## Electronic supplementary material

Below is the link to the electronic supplementary material.

Supplementary Material 1

## Data Availability

The data that support the findings of this study are not openly available due to privacy rules and for the protection of study participant privacy. Deidentified data may be made available from the corresponding author, but restrictions apply to the availability of these data.
