# Supplementary material for: Per and polyfluoroalkyl substances affect thyroid hormones for people with a history of exposure from drinking water
Source: Sci Rep. 2025 Apr 11;15:12502. doi: 10.1038/s41598-025-91977-y (PMC11992217; doi:10.1038/s41598-025-91977-y)
Supplement: Supplementary file 1 — Supplementary Material 1 [file 41598_2025_91977_MOESM1_ESM.docx]

| **Supplemental Table S1.** PFAS Analytes Limits of Quantification (LOQ) and Detection Frequencies | | | | |
| --- | --- | --- | --- | --- |
| **Abbreviation** | **Name** | **CAS Number** | **MDHHS Method LOQ (μg/L)** | **Detection Frequency**  **(% of Population)** |
| 11Cl-PF3OUdS | 11-chloroeicosafluoro-3-oxaundecane-1-sulfonate | 763051-92-9 | 0.0237 | 0.13 |
| 3:3 FTCA | 2H,2H,3H,3H-perfluorohexanoic acid (3-perfluoropropyl propanoic acid) | 356-02-5 | 0.0224 | 0.13 |
| 4:2 FTS | 1H, 1H, 2H, 2H, perfluorohexane sulfonic acid | 757124-72-4 | 0.0087 | 0.50 |
| 5:3 FTCA | 2H,2H,3H,3H-Perfluorooctanoic acid (3-perfluoropentyl propanoic acid) | 914637-49-3 | 0.0241 | 12.23 |
| 6:2 FTS | 1H, 1H, 2H, 2H, perfluorooctane sulfonic acid | 27619-97-2 | 0.0229 | 0.76 |
| 7:3 FTCA | 2H,2H,3H,3H-Perfluorodecanoic acid (3-perfluoroheptyl propanoic acid) | 812-70-4 | 0.0242 | 12.48 |
| 8:2 FTS | 1H, 1H, 2H, 2H, perfluorodecane sulfonic acid | 39108-34-4 | 0.0180 | 11.48 |
| 9Cl-PF3ONS | 9-chlorohexadecafluoro-3-oxanonane-1-sulfonate | 756426-58-1 | 0.0201 | 10.59 |
| ADONA | Dodecafluoro-3H-4,8-dioxanonanoate *or* 4,8-dioxa-3H-perfluorononanoic acid | 919005-14-4 | 0.0182 | 0.25 |
| EtFOSAA | N-Ethylperfluorooctane sulfonamidoacetic acid | 2991-50-6 | 0.0227 | 15.76 |
| HFPO-DA | Hexafluoropropylene oxide dimer acid (GenX) | 13252-13-6 | 0.0083 | 0.13 |
| NFDHA | Nonafluoro-3,6-dioxaheptanoic acid | 151772-58-6 | 0.0239 | 0.63 |
| MeFOSAA | N-Methylperfluorooctane sulfonamidoacetic acid | 2355-31-9 | 0.0132 | 94.58 |
| PFBA | Perfluorobutanoic acid | 375-22-4 | 0.0176 | 58.39 |
| PFBS | Perfluorobutanesulfonic acid | 375-73-5 | 0.0125 | 36.70 |
| PFBSA | Perfluorobutanesulfonamide | 30334-69-1 | 0.0189 | 0.13 |
| PFDA | Perfluorodecanoic acid | 335-76-2 | 0.0159 | 96.47 |
| PFDoA | Perfluorododecanoic acid | 307-55-1 | 0.0145 | 16.14 |
| PFDS | Perfluorodecanesulfonic acid | 335-77-3 | 0.0219 | 4.92 |
| PFEESA | Perfluoro (2-ethoxyethane) sulfonic acid | 113507-82-7 | 0.0178 | 1.13 |
| PFHpS | Perfluoroheptanesulfonic acid | 375-92-8 | 0.0230 | 94.70 |
| PFHxA | Perfluorohexanoic acid | 307-24-4 | 0.0113 | 3.78 |
| PFHxS | Perfluorohexanesulfonic acid | 355-46-4 | ^a^ | 100.00 |
| L-PFHxS | Perfluorohexanesulfonic acid (linear) | ^b^ | 0.0241 | 99.12 |
| Br-PFHxS | Perfluorohexanesulfonic acid (branched) | ^b^ | 0.0234 | 20.81 |
| PFHxSA | Perfluorohexanesulfonamide | 41997-13-1 | 0.0241 | 0.25 |
| PFMBA | Perfluoro-4-methoxybutanoic acid | 863090-89-5 | 0.0233 | 0.13 |
| PFMPA | Perfluoro-3-methoxypropanoic acid | 377-73-1 | 0.0221 | 0.00 |
| PFNA | Perfluorononanoic acid | 375-95-1 | 0.0235 | 98.74 |
| PFNS | Perfluorononanesulfonic acid | 68259-12-1 | 0.0198 | 0.38 |
| PFOA | Perfluorooctanoic acid | 335-67-1 | ^a^ | 100.00 |
| L-PFOA | Perfluorooctanoic acid (linear) | ^b^ | 0.0145 | 100.00 |
| Br-PFOA | Perfluorooctanoic acid (branched) | ^b^ | 0.0145 | 27.11 |
| PFOS | Perfluorooctanesulfonic acid | 1763-23-1 | ^a^ | 100.00 |
| L-PFOS | Perfluorooctanesulfonic acid (linear) | ^b^ | 0.0231 | 100.00 |
| Br-PFOS | Perfluorooctanesulfonic acid (branched) | ^b^ | 0.0239 | 100.00 |
| PFOSA | Perfluorooctanesulfonamide | 754-91-6 | 0.0192 | 0.25 |
| PFPeA | Perfluoropentanoic acid | 2706-90-3 | 0.0160 | 0.38 |
| PFPeS | Perfluoropentanesulfonic acid | 2706-91-4 | 0.0149 | 65.45 |
| PFPrS | Perfluoropropanesulfonic acid | 423-41-6 | 0.0205 | 0.25 |
| PFTeA | Perfluorotetradecanoic acid | 376-06-7 | 0.0149 | 1.89 |
| PFTriA | Perfluorotridecanoic acid | 72629-94-8 | 0.0236 | 11.98 |
| PFUnA | Perfluoroundecanoic acid | 2058-94-8 | 0.0185 | 80.58 |
| PFHpA | Perfluoroheptanoic acid | 375-85-9 | 0.0167 | 67.47 |
| PFECHS | Perfluoroethylcyclohexane sulfonate | 646-83-3 | 0.0223 | 76.92 |
| ^a^Calculated values (i.e., parents of isomers) do not have LOQs.  ^b^CAS numbers for Br- and Ln- isomers are not reported. | | | | |

**Supplemental Table S2. Thyroid hormone reference ranges.**

| **Thyroid Hormone** | **MDHHS BOL**  **Reference Range (Adult)** | **Mayo Clinic Laboratories**  **Reference Range (Adult)** |
| --- | --- | --- |
| Thyroid Stimulating Hormone (TSH)^1^ | 0.358-3.74 ulU/mL | n/a |
| Total Thyroxine (T4)^1^ | Males: 4.5-12.1 ug/dL  Females: 4.8-13.9 ug/dL | n/a |
| Free T4^1^ | 13-20 years: 0.78-1.34 ng/dL  18-59 years: 0.76-1.46 ng/dL | n/a |
| Total Triiodothyronine^1,2^ | 13-19 years: 86-192 ng/dL  ≥ 20 years: 60-181 ng/dL | 11-19 years: 91-218 ng/dL  ≥ 20 years: 80-200 ng/dL |

**Table notes.** n/a= not applicable. Adapted from MDHHS Bureau of Laboratories Report, *MiPEHS Biomarker Reference Ranges* (2020)

2<https://www.mayomedicallaboratories.com/test-catalog/Clinical+and+Interpretive/8613>

| **Table S3. Univariate Linear Regression** Adjusted^a^ Change in Thyroid Hormones^b^ per 1% Increase in Serum PFAS Concentration among MiPEHS Participants^c^ for PFAS Detected in at Least 60% of Participants | | | | | | | | |
| --- | --- | --- | --- | --- | --- | --- | --- | --- |
| PFAS Analytes^d^ |  | Percent change (95% CI)  in TT3 (n=684) |  | Percent change (95% CI) in fT4 (n=727) |  | Percent change (95% CI) in TSH (n=726) |  | Absolute change (95% CI) in TT4 (n=728) |
| MeFOSAA |  | 0.01 (0,0.03) |  | 0.01 (0,0.02) |  | -0.03 (-0.07,0.01) |  | 0 (0,0) |
| PFDA |  | -0.019 (-0.03,0) |  | -0.004 (-0.02,0.01) |  | 0.07 (0.01,0.12) |  | 0 (0,0) |
| PFECHS |  | -0.015 (-0.03,0) |  | 0 (-0.01,0.01) |  | 0.01 (-0.04,0.07) |  | -0.001 (0,0) |
| PFHpA |  | 0.01 (0,0.02) |  | -0.01 (-0.02,0) |  | 0.06 (0.02,0.11) |  | 0 (0,0) |
| PFHpS |  | 0.004 (0,0.01) |  | 0.003 (0,0.01) |  | -0.01 (-0.04,0.02) |  | 0 (0,0) |
| PFNA |  | -0.014 (-0.03,0) |  | 0.002 (-0.01,0.01) |  | 0.02 (-0.03,0.08) |  | 0 (0,0) |
| PFPeS |  | -0.01 (-0.02,0) |  | 0 (-0.01,0) |  | 0.02 (-0.02,0.06) |  | 0 (0,0) |
| PFUnA |  | -0.023 (-0.04,-0.01)* |  | 0.001 (-0.01,0.01) |  | 0.05 (0,0.1) |  | 0 (0,0) |
| Br_PFOS |  | 0.005 (0,0.02) |  | 0.003 (0,0.01) |  | -0.01 (-0.05,0.02) |  | 0 (0,0) |
| Ln_PFOS |  | -0.003 (-0.02,0.01) |  | 0 (-0.01,0.01) |  | 0.01 (-0.04,0.05) |  | -0.001 (0,0) |
| PFHxS |  | 0 (-0.01,0.01) |  | 0.01 (0,0.01) |  | -0.01 (-0.05,0.02) |  | 0 (0,0) |
| PFOA |  | 0.002 (-0.01,0.01) |  | 0 (-0.01,0.01) |  | -0.02 (-0.04,0.01) |  | 0 (0,0) |
| Beta estimates were back-transformed for interpretation. | | | | | | | | |
| *Significant at adjusted P-value <0.05 (after the Benjamini and Hochberg (BH) correction for multiple comparisons) | | | | | | | | |
| ^a^Adjusted for covariates determined a priori: sex, age at blood draw, smoking status, recent alcohol use, and family history of thyroid disease. | | | | | | | | |
| ^b^Thyroid Stimulating Hormone (TSH); Total Triiodothyronine (TT3); Free Thyroxine (fT4); Total Thyroxine (TT4) | | | | | | | | |
| ^c^Participants who did not self-report taking a thyroid-altering medication in the 30 days prior to their survey  ^d^N-Methylperfluorooctane sulfonamidoacetic acid (MeFOSAA); Perfluorodecanoic acid (PFDA); Perfluoroethylcyclohexane sulfonate (PFECHS); Perfluoroheptanoic acid (PFHpA); Perfluoroheptanesulfonic acid (PFHpS);Perfluorohexanesulfonic acid (branched and linear) (PFHxS); Perfluorononanoic acid (PFNA); Perfluorooctanoic acid (branched and linear) (PFOA); Perfluorooctanesulfonic acid (branched and linear) (PFOS); Perfluoropentanesulfonic acid (PFPeS); Perfluoroundecanoic acid (PFUnA) | | | | | | | | |

| **Supplemental Table S4.** Loading factors for each substance on the first two components obtained by supervised principal component analysis | | | | | | | | |
| --- | --- | --- | --- | --- | --- | --- | --- | --- |
|  | **Component 1** | | | | **Component 2** | | | |
| **PFAS^a^** | **TSH^a^ (n=726)** | **TT4 (n=728)** | **fT4^a^**  **(n=727)** | **TT3^a^**  **(n=684)** | **TSH^a^**  **(n=726)** | **TT4 (n=728)** | **fT4^a^**  **(n=727)** | **TT3^a^**  **(n=684)** |
| MeFOSAA | 0.00 | 0.00 | -0.19 | 0.00 | 0.00 | 0.00 | -0.96 | 0.00 |
| PFDA | -0.69* | -0.54* | 0.00 | -0.55* | 0.14 | -0.18 | 0.00 | 0.31 |
| PFECHS | 0.00 | -0.44 | 0.00 | -0.38 | 0.00 | 0.08 | 0.00 | -0.86 |
| PFHpA | -0.21 | -0.17 | 0.00 | 0.00 | -0.98 | 0.95 | 0.00 | 0.00 |
| PFHpS | 0.00 | 0.00 | -0.62* | 0.00 | 0.00 | 0.00 | 0.03 | 0.00 |
| PFNA | -0.56* | 0.00 | 0.00 | -0.54* | -0.06 | 0.00 | 0.00 | -0.07 |
| PFPeS | 0.00 | 0.00 | -0.46* | 0.00 | 0.00 | 0.00 | 0.28 | 0.00 |
| PFUnA | -0.68* | -0.51* | 0.00 | -0.51* | 0.17 | -0.22 | 0.00 | 0.39 |
| PFHxS | 0.00 | 0.00 | 0.00 | 0.00 | 0.00 | 0.00 | 0.00 | 0.00 |
| PFOA | 0.00 | 0.00 | 0.00 | 0.00 | 0.00 | 0.00 | 0.00 | 0.00 |
| Ln_PFOS | 0.00 | -0.48* | 0.00 | 0.00 | 0.00 | 0.03 | 0.00 | 0.00 |
| Br_PFOS | 0.00 | 0.00 | -0.60* | 0.00 | 0.00 | 0.00 | 0.05 | 0.00 |
| ^a^Log-transformed. “*” indicates loadings over 0.50. | | | | | | | | |

|  |
| --- |

| **Supplemental Table S5**. Conditional posterior inclusion probabilities (PIP) of PFAS (n=11) for each thyroid hormone. | | | | |
| --- | --- | --- | --- | --- |
| **PFAS^a^** | **TSH^a^**  **(n=726)** | **TT4**  **(n=728)** | **fT4^a^**  **(n=727)** | **TT3^a^**  **(n=684)** |
| MeFOSAA | 0.036 | 0.032* | 0.648* | 0 |
| PFDA | 0.106* | 0.025* | 0.442 | 0.006 |
| PFECHS | 0.017 | 0 | 0.408 | 0 |
| PFHpA | 0.184* | 0.001 | 0.694* | 0 |
| PFNA | 0.028 | 0.035* | 0.454 | 0 |
| PFPeS | 0.037 | 0.002 | 0.483 | 0.013 |
| PFUnA | 0.043* | 0.005 | 0.440 | 0.051 |
| PFHxS | 0.028 | 0 | 0.523* | 0 |
| PFOA | 0.024 | 0 | 0.366 | 0 |
| Br_PFOS | 0.016 | 0.003 | 0.368 | 0 |
| Ln_PFOS | 0.020 | 0.024 | 0.412 | 0 |
| ^a^Log-transformed, “*” indicates highest three PIPs within each thyroid hormone. | | | | |

# Supplemental Figure S1. Pearson correlation matrix between (log) PFAS, including branched and linear isomers of PFOS, among all participants (n=728).


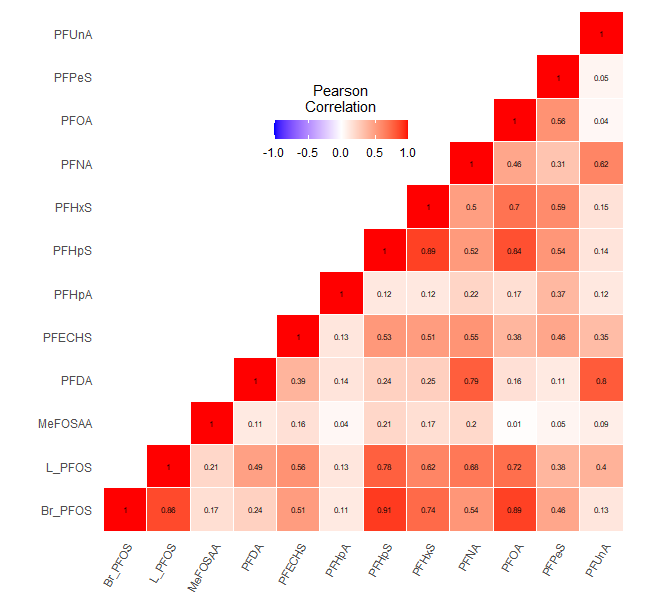


# Supplemental Figure S2. Weights of PFAS used in Weighted Quantile Sum Regression for four thyroid hormones assuming a negative relationship (Panel A) and a positive relationship (Panel B).


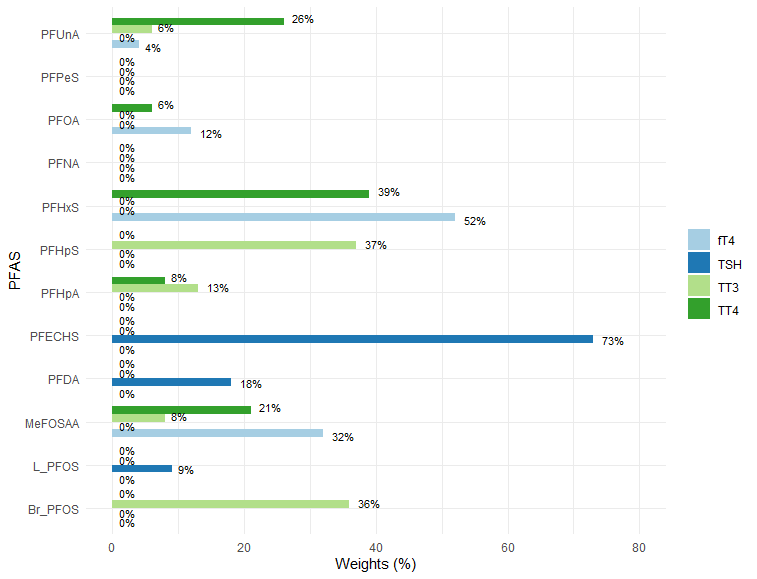


**B**


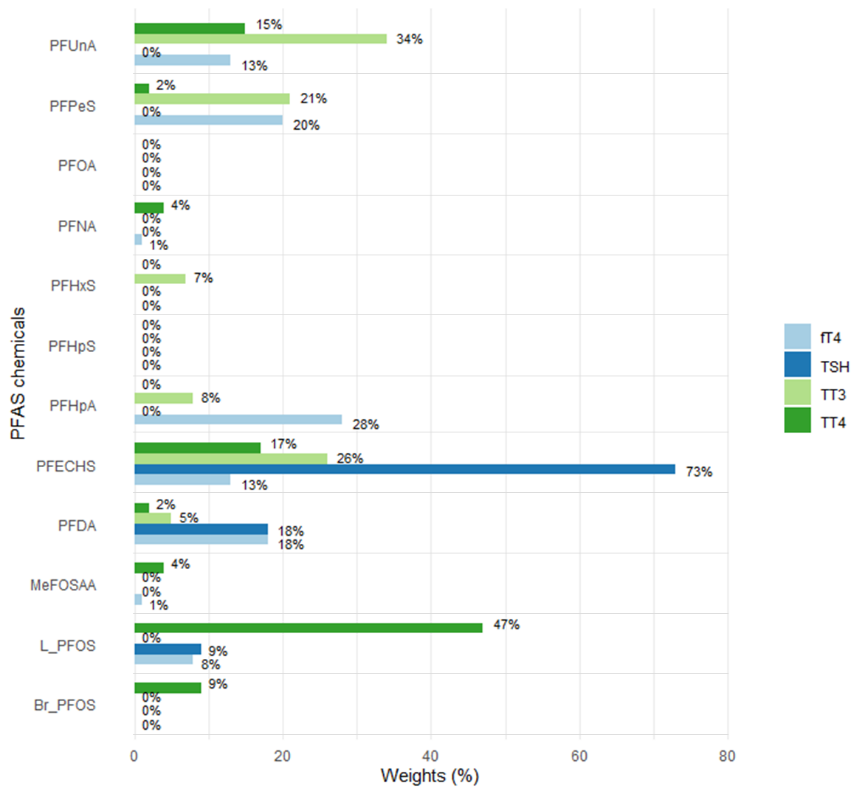


**A**

# Supplemental Figure S3. Exposure-response functions in BKMR for FT4 (panel A), TT4 (panel B), TT3 (panel C), TSH (panel D).

# Panel A. Outcome: FT4


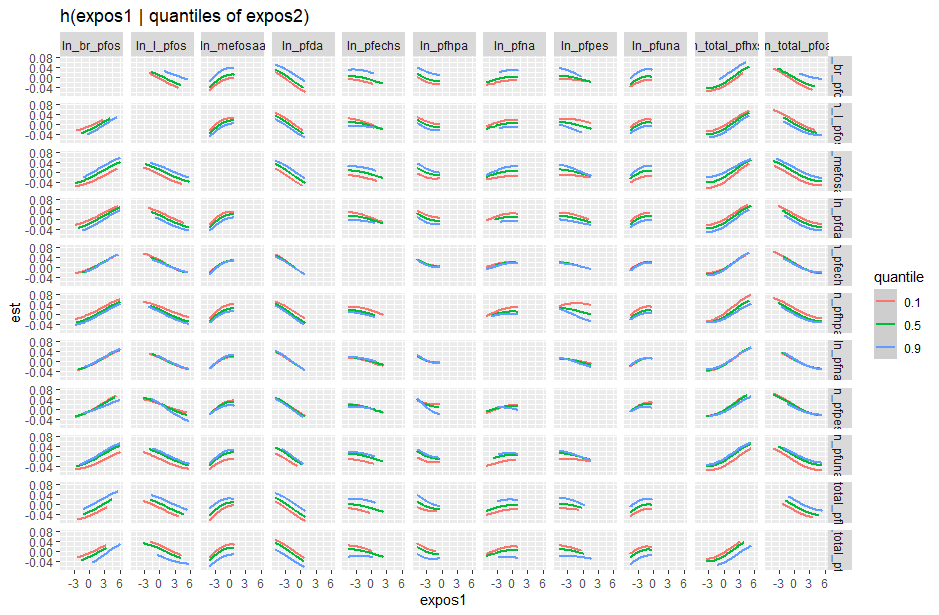


PFAS chemicals (log transformed)

Estimate

**Panel B. Outcome: TT4**

Estimate


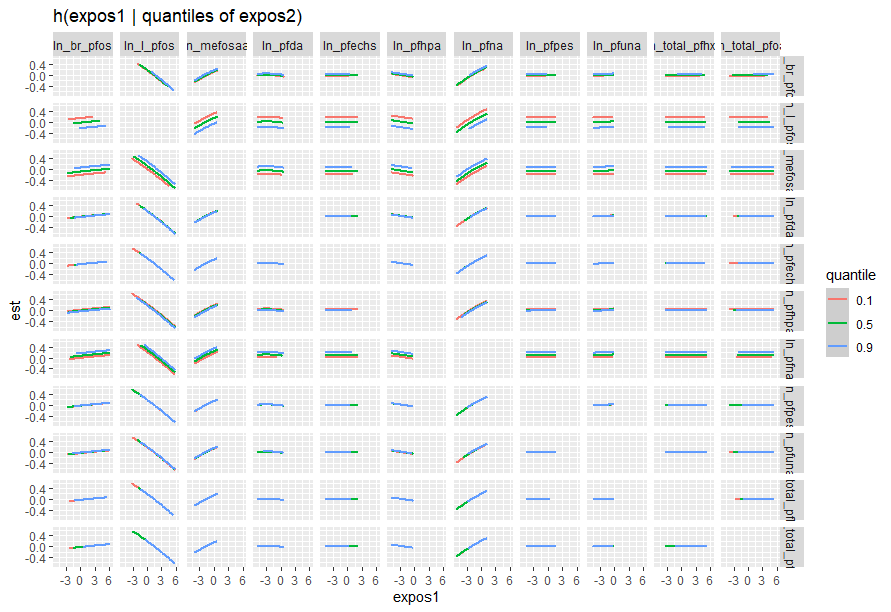


PFAS chemicals (log transformed)

**Panel C. Outcome: TT3**


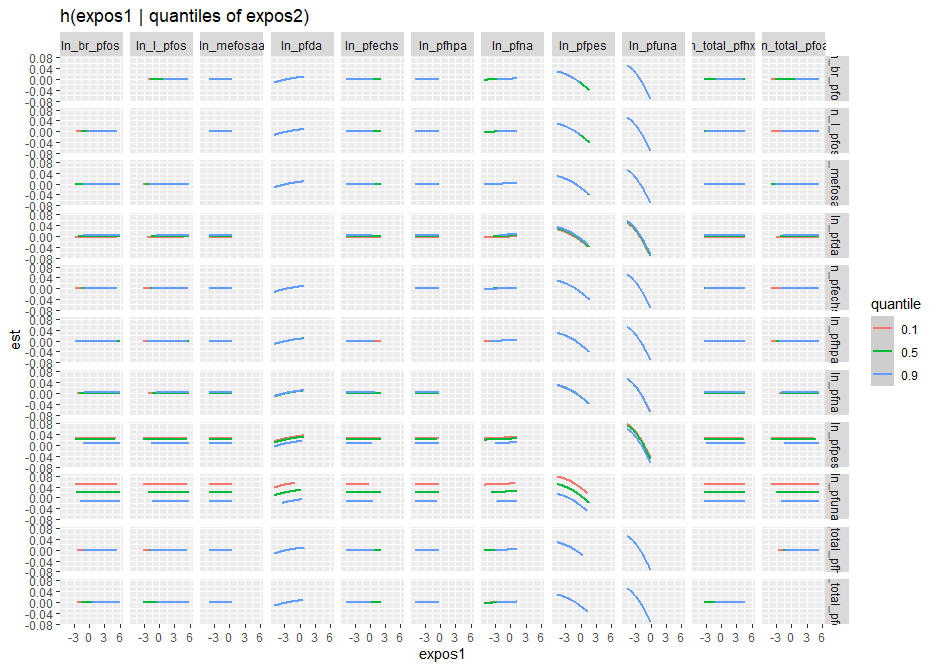


PFAS chemicals (log transformed)

Estimate

PFAS chemicals (log transformed)

**Panel D. Outcome: TSH**

#


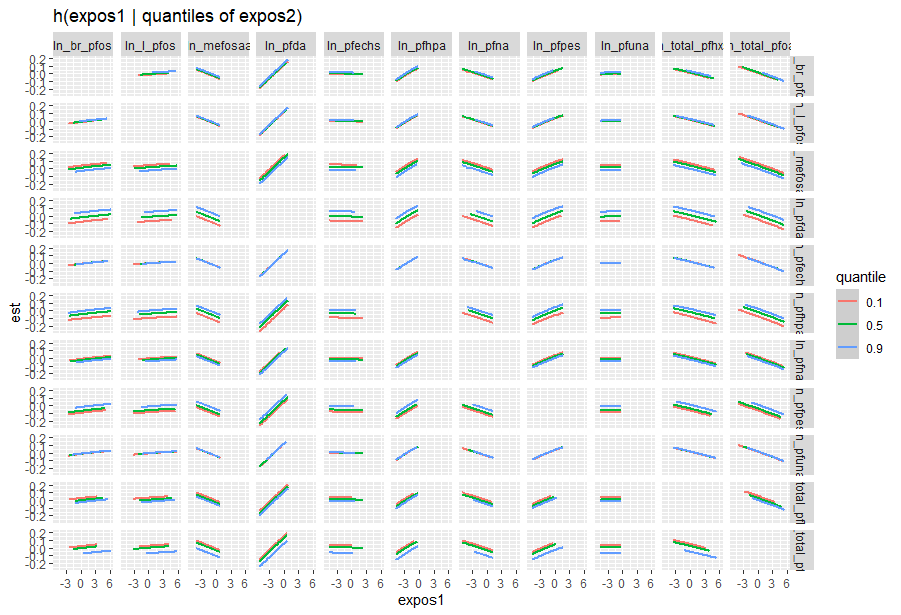


PFAS chemicals (log transformed)

Estimate

Notes: Exposure response functions between exposure to each PFAS and four Thyroid functions while each other PFAS is fixed at defined percentiles (10th, 50th, and 90th), all other substances are fixed at their median value. “ln” refers to the log transformation performed. “Br” refers to the branched isomers of the PFAS. “L” refers to the linear isomers of the PFAS. Note Br and L isomers are only available separately for PFOS, PFOA, PFHxS.
